# P-654. Demographic and Clinical Characteristics, by Pneumococcal Serotype, of Adults Hospitalized with Community Acquired Pneumonia in the United States

**DOI:** 10.1093/ofid/ofaf695.867

**Published:** 2026-01-11

**Authors:** Lindsay Grant, Jelena Vojicic, Mohammad Ali, Paul Palmer, Alejandro D Cane, Michael Bois, Paula Peyrani

**Affiliations:** Pfizer Inc., Collegeville, PA; Pfizer Canada, Kirkland, QC, Canada; Pfizer Inc., Collegeville, PA; Pfizer Vaccine Medical Development, Scientific & Clinical Affairs , Collegeville PA, Collegeville, PA; Pfizer, Collegeville, Pennsylvania; Pfizer, Collegeville, Pennsylvania; Pfizer, Inc, Collegeville, PA

## Abstract

**Background:**

Characteristics of community acquired pneumonia (CAP) due to Streptococcus pneumoniae could differ based on the capsular serotype, which has been associated with virulence and invasiveness. The objective of this study was to describe the demographic and clinical characteristics of CAP cases by serotype, for serotypes included in the 20-valent pneumococcal conjugate vaccine (PCV20).

**Table.**
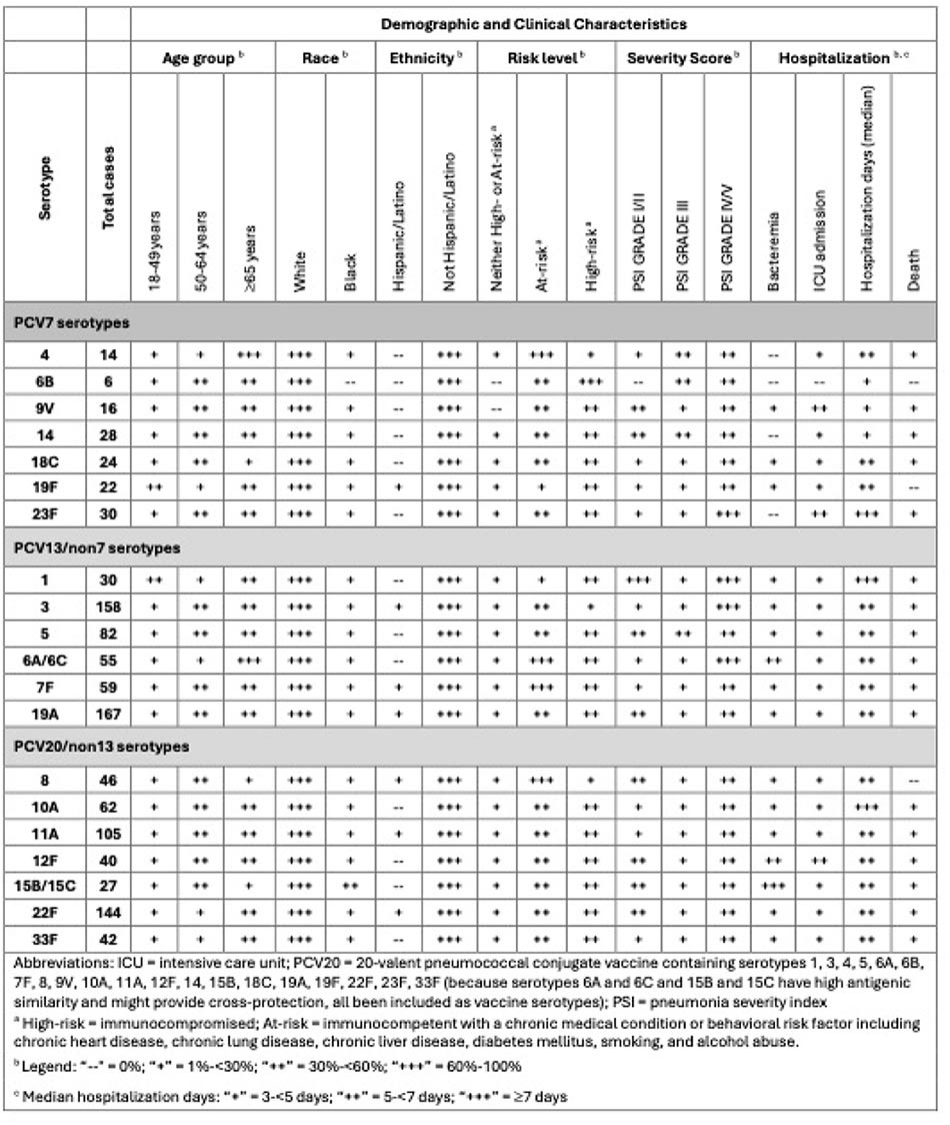
Classification of serotype-specific CAP by demographic and clinical characteristics

**Methods:**

We analyzed previously collected data from adults ≥18 years of age hospitalized with CAP from 2014-2016 and 2019-2020 at multiple US sites. Serotyping was performed from cultures of blood and respiratory specimens, and by Pfizer’s serotype-specific urinary antigen detection assay. Demographics and clinical history were abstracted from the medical chart. Within each serotype, distribution of cases with characteristics of interest was determined (Table).

**Results:**

The most common PCV20 serotypes were 19A, 3, 22F, 11A and 5. Cases with the highest proportion of severe outcomes were due to serotypes 1, 3, 6A/6C and 23F (PSI Grade IV/V) and 9V, 12F and 23F (ICU admission); all serotypes but 6B, 8 and 19F were associated with mortality. Cases of CAP due to serotype 6A/C, 12F and particularly 15B/C had higher proportions of bacteremia, and those with serotypes 1, 10A and 23F had the longest hospital stay. Compared to other serotypes, cases of 19F and 1 were enriched for those aged 18-49 years, and 4 and 6A/6C for those aged ≥65 years, while 15B/C cases occurred proportionally more among Black people.

**Conclusion:**

Certain serotypes caused more severe disease, while being of relatively lower prevalence. Additional research considering serotype-specific disease outcomes and host factors may further inform the choice of PCV.

**Disclosures:**

Lindsay Grant, PhD, MPH, Pfizer: Employee|Pfizer: Stocks/Bonds (Private Company) Jelena Vojicic, MD, Pfizer Inc.: Employee|Pfizer Inc.: Stocks/Bonds (Public Company)|Pfizer Inc.: Stocks/Bonds (Public Company) Mohammad Ali, PhD, Pfizer Vaccines: Employee|Pfizer Vaccines: Stocks/Bonds (Public Company) Paul Palmer, PhD, Pfizer Inc: Employee|Pfizer Inc: Stocks/Bonds (Public Company) Alejandro D. Cane, MD, PhD, Pfizer Inc.: All authors are employees of Pfizer Inc. and may hold stock and/or stock options of Pfizer Inc. Michael Bois, PhD, M(ASCP), PFIZER: Stocks/Bonds (Public Company) Paula Peyrani, MD, Pfizer, Inc: Employee|Pfizer, Inc: Stocks/Bonds (Public Company)

